# Association of Sex With Adolescent Soccer Concussion Incidence and Characteristics

**DOI:** 10.1001/jamanetworkopen.2021.8191

**Published:** 2021-04-27

**Authors:** Abigail C. Bretzin, Tracey Covassin, Douglas J. Wiebe, William Stewart

**Affiliations:** 1 Penn Injury Science Center, Department of Biostatistics, Epidemiology, and Informatics, Perelman School of Medicine, University of Pennsylvania, Philadelphia; 2 Department of Kinesiology, Michigan State University, East Lansing; 3 Department of Neuropathology, Queen Elizabeth University Hospital, Glasgow, United Kingdom; 4 Institute of Neuroscience and Psychology, University of Glasgow, Glasgow, United Kingdom

## Abstract

**Importance:**

Because of the negative consequences of concussion, considerable research efforts have been directed toward understanding the risk factors for sport-related concussion (SRC) and its outcomes to better inform strategies for risk reduction. Girls are suggested to have an increased risk of concussion, warranting exploration into sex-dependent variations in concussion presentation and management, with the potential that this information might inform sex-specific rules directed toward risk reduction within sports.

**Objective:**

To compare sex-associated differences in epidemiology and concussion management in adolescent soccer players within a prospective, longitudinal high school injury surveillance project.

**Design, Setting, and Participants:**

This prospective, longitudinal cohort study assessed male and female soccer athletes from all high schools in the Michigan High School Athletic Association (MHSAA) during academic years 2016-2017 to 2018-2019.

**Exposures:**

Sport-related concussion captured in the MHSAA Head Injury Reporting System.

**Main Outcomes and Measures:**

Outcomes included details regarding each documented SRC event, including injury mechanism, immediate management, and return-to-play time. Multiple comparisons were made between male and female athletes regarding SRC risk, mechanism, short-term management, and outcomes.

**Results:**

A total of 43 741 male and 39 637 female soccer athletes participated in MHSAA soccer during the 3 consecutive academic years of study (2016-2017: n = 751 schools; 2017-2018: n = 750 schools; and 2018-2019: n = 747 schools). During the 3 years of surveillance, 1507 of the 83 378 soccer athletes (1.8%) were reported to have SRC during soccer participation, including 557 boys (37.0%) and 950 girls (63.0%). Documented SRC risk in female soccer participants was greater than in male soccer participants (risk ratio, 1.88; 95% CI, 1.69-2.09; *P* < .001). Male soccer athletes most often sustained SRC from contact with another player (48.4%), whereas SRCs in female soccer players recorded in the Head Injury Reporting System were most often from nonplayer contact events (41.9%; *P* < .001). Adolescent male soccer players with a documented SRC were more likely to be removed from play on the day of injury (odds ratio, 1.54; 95% CI, 1.15-2.06; *P* = .004). Although the overall median time to return to play was 11 days (interquartile range [IQR], 7-15 days), male athletes typically returned 2 days earlier than female athletes (median, 10 [IQR, 7-14] days vs 12 [IQR, 7-16] days; Peto test *P* < .001).

**Conclusions and Relevance:**

In this cohort study, sex-associated differences were revealed among adolescent soccer athletes in SRC risk, mechanism of injury, immediate management, and outcomes in injuries documented in a statewide injury reporting system. Thus, consideration might be given to sex-specific approaches to participation and concussion management in the sport.

## Key Points

**Question:**

Do sex-associated differences exist in sport-related concussion (SRC) risk, mechanism, management, and recovery in adolescent soccer?

**Findings:**

In this cohort study of a high school injury surveillance project, the risk of documented SRC was 1.88 times higher among adolescent girls than adolescent boys. Whereas boys most often sustained SRC from player contact (48.4%), documented SRCs in girls were most often from equipment contact (eg, ball or goalpost [41.9%]), and boys had 1.54 greater odds of immediate removal from play and returned to play 2 days sooner than girls.

**Meaning:**

These findings suggest that concussion risk and outcome differences in adolescent soccer athletes might require sex-specific approaches to participation and concussion management in sport.

## Teaser

This cohort study compares sex-associated differences in epidemiologic factors and concussion management in adolescent soccer players within a high school injury surveillance project.

## Introduction

Concern exists regarding the immediate and late consequences of sport-related concussion (SRC).^1,2,3^ In part, this concern reflects increasing awareness of the proposed association between the history of SRC and repetitive head impacts and increased risk of neurodegenerative disease, including chronic traumatic encephalopathy and Alzheimer disease.^3,4,5^ Therefore, considerable research efforts have been directed toward understanding of the risk factors for SRC and its outcomes to better inform strategies for risk reduction. Multiple studies^6,7,8,9,10,11,12^ have suggested that female athletes are at increased SRC risk compared with male athletes in sports in which rules are comparable. However, limited understanding remains regarding why SRC risk in female athletes appears increased. In this context, sex-dependent variations in concussion presentation and management need to be explored, with the potential that this information might inform sex-specific rules directed to risk reduction within sports.

Surveillance studies in the Ivy League^10^ and more broadly across US athletic conferences^8^ report an elevated risk of SRC among female collegiate athletes compared with male athletes. Similarly, female soccer athletes have a disproportionately higher risk of SRC in statewide and national high school injury surveillance studies.^6,9,11^ Furthermore, female soccer athletes are suggested to exhibit longer recovery outcomes.^12^ Notably, previous work^6,8,9,10,11^ exploring differences between adolescent female and male soccer athletes is limited in consideration of the role of game factors and SRC management on risk and outcomes, which might inform potential prevention initiatives within the sport.

Soccer is the world’s most popular participation sport, with an estimated 250 million active participants globally.^13^ Compared with many contact sports, risk of SRC and more severe traumatic brain injuries in soccer is low.^14^ Nevertheless, there is concern around potential lifelong consequences of exposure to head impacts and traumatic brain injury in soccer.^15,16^ Of importance, however, although there are high participation rates in youth and adolescent soccer programs and most participants are at the amateur level, to date much of the data on SRC and outcomes in this sport derive from observations in adult and elite athletes.

To better understand the factors that contribute to differences between male and female athletes with respect to SRC in soccer, we conducted a statewide, prospective cohort study to gather information on injury outcomes in adolescent (high school) athletes. We then interrogated the collated data on sex differences in reported SRC incidence, mechanism of injury, immediate management, and outcomes.

## Methods

### Research Participants

All high school (9th grade through 12th grade) soccer athletes in Michigan from the beginning of academic year 2016-2017 to the end of academic year 2018-2019 were included in this study. The Michigan High School Athletic Association (MHSAA) Head Injury Reporting System mandates that, since the 2015-2016 academic year, all high schools in the state of Michigan enter data in the MHSAA Head Injury Reporting System. In addition to data on all reported SRCs, the Head Injury Reporting System includes the total number of participating athletes in each sport. The Michigan State University Institutional Review Board approved this study as exempt because of the use of deidentified data; therefore, no informed consent was required. The study followed the Strengthening the Reporting of Observational Studies in Epidemiology (STROBE) reporting guideline.^17^

### Data Reporting and Definitions

The MHSAA requires each recognized SRC to be recorded via an online repository. Athletic trainers were the primary data recorders. If an athletic trainer was not employed by a high school, coaches and school officials were required to report data into the Head Injury Reporting System. The demographic data include sex, grade, level of competition (freshman, junior varsity, or varsity), and sport. Athletic trainers or other school officials reported sex based on whether the athlete was participating on a boys’ or girls’ team. The SRC injury event data include injury, initial evaluator, whether the SRC occurred in a game or practice, mechanism of injury, and individual who authorized return-to-play clearance. Each student athlete was provided a 7-digit identification number to ensure that the data remained anonymous. If a student athlete sustained subsequent SRCs, each SRC was recorded as a separate injury.

The MHSAA staff monitored data entry daily for errors. If an error was identified, schools were contacted to make edits and corrections. The MHSAA deemed SRC data collection complete when no less than 99% of all injury reports were received from each school. Only MHSAA staff members were authorized to edit SRC reports once submitted. Further details on the MSHSAA Head Injury Reporting System can be found in previously published studies.^9,18^

### Operational Definitions

#### Sport-Related Concussion

The MHSAA defined SRC as (1) an injury occurring as a result of participation during preseason, in-season, and postseason practice, scrimmage, or game for MHSAA-sanctioned sports that provided a postseason tournament and (2) required the student athlete to be withheld from activity after exhibiting signs, symptoms, or behaviors consistent with an SRC. All SRCs required confirmation of diagnosis by a physician, osteopath, nurse practitioner, or physician assistant.

#### Initial Examiner

The MHSAA Head Injury Reporting System requires that the personnel assessing the student athlete with suspected SRC at the time of injury and responsible for the decision on immediate management is recorded. For the purposes of this study, the initial examiner was coded as either athletic trainer or not athletic trainer. Certified athletic trainers are allied health care professionals involved in the prevention, examination, diagnosis, treatment, and rehabilitation of emergency, acute, or chronic injuries and medical conditions. The Athletic Training Locations and Services Annual Report^19^ identified that, in Michigan, 52.3% of high schools had athletic training services, with variable access to full-time or part-time athletic training services.

#### Mechanism of Injury

The MHSAA captured data on mechanisms of injury under 4 possible categories: person-to-person contact, person-to-object contact, person-to-playing surface contact, or uncertain about the cause of the event. Person-to-object contact involved contact with anything other than the playing surface or person, which in soccer included equipment such as, but not limited to, the soccer ball and goalposts.

#### Removal From Activity

For each documented SRC, data on whether the athlete was removed from activity at the time of injury were recorded. If the SRC was not recognized or reported at the time of injury, this was recorded as not removed from activity.

#### Return-to-Play Clearance

The MHSAA requires medical clearance in writing and was only recorded for unrestricted return to full participation. Only a physician, osteopath, nurse practitioner, or physician assistant is authorized to clear a concussed soccer athlete for unrestricted activity.

### Statistical Analysis

Statistical analyses were performed using StataCorp software, version 16.0 (StataCorp LLC). Between-group differences in categorical data were compared using χ^2^ tests; missing data were reported and managed using listwise deletion. Differences in the incidence proportion of reported SRC between male and female athletes were expressed as relative risks (RRs) with 95% CIs. Logistic regression analysis was used to estimate the odds ratios (ORs) and 95% CIs of removal from activity comparing sex of participants and involvement of an athletic trainer and comparing the interaction between these variables. Descriptive statistics present the median and interquartile range (IQR) for time to return to play in days. Kaplan-Meier survival with Peto analyses were used to test for difference in return-to-play time between the sexes. Significance was set a priori at a 2-sided *P* < .05.

## Results

### Concussion Risk and Mechanisms in Adolescent Soccer

A total of 43 741 male and 39 637 female soccer athletes participated in MHSAA soccer during the 3 consecutive academic years of study. During this time, the MHSAA Head Injury Reporting System recorded 1507 soccer-related SRCs (37.0% male). A greater proportion of female athletes than male athletes were in lower grades in school (308 [32.4%] vs 117 [21.0%] in ninth grade; *P* < .001), participated in junior varsity soccer (337 [35.5%] vs 141 [27.1%]; *P* = .004), or had a prior history of concussion (199 [21.0%] vs 78 [14.0%]; *P* < .001) (Table 1). Concussion history was missing for 5 female athletes (0.5%). Most documented SRC occurred during competition in both male (813 [85.5%]) and female (476 [85.6%]) athletes (*P* = .55). The overall incidence of documented SRC was 1.8 (95% CI, 1.72-1.90) per 100 athletes per season. Across all academic years, female soccer athletes had a higher risk of SRC than their male counterparts (RR, 1.88; 95% CI, 1.69-2.09; *P* < .001) (Table 2).

**Table 1.** Student Athlete Characteristics for Sport Related Concussion

Characteristic	No. (%) of athletes		*P* value^a^
	Male	Female	
Total	557 (37.0)	950 (63.0)	
Grade			
9th	117 (21.0)	308 (32.4)	<.001
10th	152 (27.3)	272 (28.6)	
11th	140 (25.1)	213 (22.4)	
12th	148 (26.6)	157 (16.5)	
Level of competition			
Freshman	15 (2.7)	23 (2.4)	.004
Junior varsity	151 (27.1)	337 (35.5)	
Varsity	391 (70.2)	590 (62.1)	
History of previous concussion			<.001
Yes	78 (14.0)	199 (21.0)	
No	479 (86.0)	746 (78.5)	
Missing	0	5 (0.5)	
Activity			
Practice	81 (14.5)	137 (14.4)	.55
Competition	476 (85.5)	813 (85.6)	

^a^ χ^2^ test.

**Table 2.** Incidence of Sport-Related Concussion in Soccer by Academic Year

Academic year	No. of concussions	No. of participants	RR (95% CI)	*P* value^a^
2016-2017				
Female athletes	349	13 212	1.89 (1.58-2.25)	<.001
Male athletes	205	14 630	1 [Reference]	
2017-2018				
Female athletes	305	13 216	2.07 (1.71-2.52)	<.001
Male athletes	163	14 619	1 [Reference]	
2018-2019				
Female athletes	296	13 209	1.72 (1.43-2.07)	<.001
Male athletes	189	14 492	1 [Reference]	
Total				
Female athletes	950	39637	1.88 (1.69-2.09)	<.001
Male athletes	557	43741	1 [Reference]	

Abbreviation: RR, risk ratio.

^a^ Incidence rate ratio.

Information on mechanism of injury was available for all but 11 reported SRCs (0.7%). Overall, the most common mechanisms of injury were contact with an object (571 [38.2%]) or with another player (567 [37.9%]). However, the distribution of injury mechanisms was not consistent between male and female athletes, with the most common mechanism cited for documented SRC in female athletes being contact with an object (398 [41.9%]), whereas in male athletes the most common mechanism of injury was contact with another player (264 [48.4%]; *P* < .001) (Table 3).

**Table 3.** Mechanism of Injury for Sport-Related Concussion

Mechanism of injury	No. (%) of athletes			*P* value^b^
	Total	Male^a^	Female	
Contact with object	571 (38.2)	173 (31.7)	398 (41.9)	<.001
Contact with person	567 (37.9)	264 (48.4)	303 (31.9)	
Contact with playing surface	233 (15.6)	67 (12.3)	166 (17.5)	
Uncertain	125 (8.4)	42 (7.7)	83 (8.7)	

^a^ Mechanism of injury data missing for 11 male athletes with sport-related concussion.

^b^ χ^2^ test.

### Immediate Concussion Management and Return to Play in Adolescent Soccer

Overall, 1237 of 1507 reported SRCs (82.1%) among adolescent soccer athletes led to removal from activity on the day of injury, with 1078 of 1507 SRCs (71.5%) assessed by an athletic trainer as at least 1 of the personnel involved in their initial evaluation. However, removal from activity was not consistent between male and female athletes, with male athletes more likely to be removed from play than female athletes (OR, 1.54; 95% CI, 1.15-2.06; *P* = .004). Where athletic trainers were involved in the initial evaluation, athletes with documented SRC were more likely to be removed from activity than when no athletic trainer was involved (OR, 3.12; 95% CI, 2.38-4.11; *P* < .001). There was no interaction between male or female sex and athletic trainer or no athletic trainer involvement and removal from play (OR, 0.69; 95% CI, 0.38-1.24; *P* = .38).

By the end of data collection, 1247 of 1507 adolescent soccer athletes (82.8%) with documented SRC obtained clearance to return to full participation, with male athletes (486 [87.3%]) more likely to be cleared for return to play than female athletes (761 [80.1%]; *P* < .001). Furthermore, although the overall median time to return to play was 11 days (IQR, 7-15 days), male athletes typically returned 2 days earlier than female athletes (Peto test *P* < .001) (Figure). Although no difference in return-to-play time was found between female athletes with a history of previous concussion vs those without (median, 11 [IQR, 7-18] days vs 12 [IQR, 7-16] days; Peto test *P* = 0.93), male athletes with a history of previous concussion took a median of 2 days longer to return-to-play time than those without (median, 11 [IQR, 8-15] days vs 9 [IQR, 7-14] days; Peto test *P* = .02) (eFigure in the Supplement).

**Figure.**
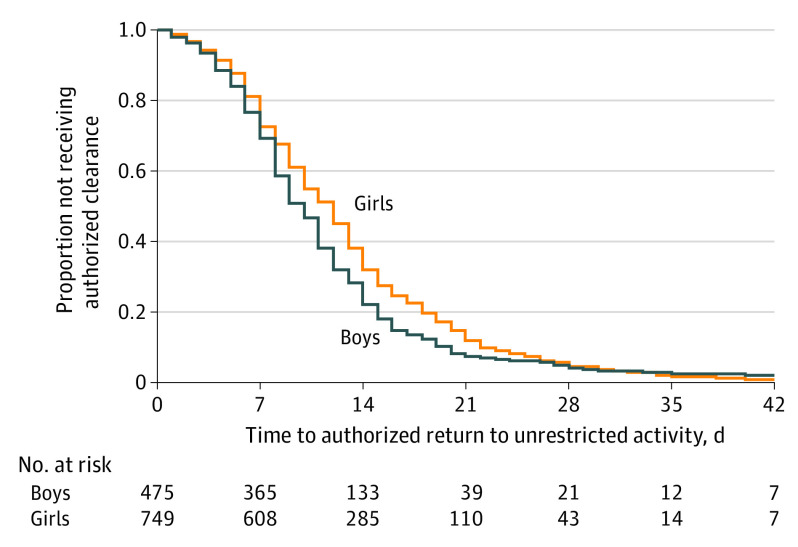
Time to Authorized Return to Unrestricted Activity Kaplan-Meier survival curve presenting the proportion of adolescent soccer athletes not authorized clearance to return to unrestricted activity. Median time to return to activity was 10 days (interquartile range [IQR], 7-14 days) in male athletes and 12 days (IQR, 7-16 days) in female athletes (Peto test *P* < .001).

## Discussion

This cohort study provides clear evidence of sex-associated differences in concussion risk, mechanism of injury, short-term management, and time to unrestricted return to play after injury among adolescent soccer athletes. Specifically, female adolescent soccer athletes had a greater risk of documented SRC and took a median of 2 days longer to return to play compared with male athletes. Furthermore, a greater proportion of female than male athletes sustained SRC from contact with an object, with male athletes more likely to sustain injury from contact with another player. Finally, immediate removal from play was more common for adolescent male soccer athletes and when an athletic trainer was involved in the immediate evaluation of athletes with suspected SRC.

### Sex Differences in Concussion Risk

The observation that female adolescents participating in soccer had a greater risk of documented SRC compared with male adolescents is in line with studies^9,20^ that have identified sex differences in SRC risk. For example, at the high school level, female soccer athletes have an approximately 60% to 80% greater risk of SRC than male athletes.^9,20^ Although multiple studies^6,8,9,10,11^ report increased concussion risk in female athletes, reasons for this increased risk remain unclear. In part, this finding might reflect greater likelihood of symptom reporting in female athletes.^21^ Alternatively, variation in SRC risk between the sexes may be a consequence of physiological differences between male and female athletes contributing to concussion propensity. Female soccer athletes have lower neck strength and girth compared with male athletes,^22,23,24,25^ with these variables inversely associated with linear and rotational head acceleration after soccer ball heading.^24,25^ At the cellular level, axons in female individuals have smaller and fewer microtubules than those in male individuals, predisposing them to greater risk of injury under dynamic stretch.^26^ Thus, as a result of anthropometric and brain microstructural differences between the sexes, female athletes may be at greater risk than male athletes of diffuse axonal injury, the principal pathology underlying concussion.^3^

### Sex Differences in Concussion Mechanism in Adolescent Soccer

The most common mechanisms for concussion reported in this study were contact with an object or another player. Intriguingly, however, there was a sex difference in concussion mechanism between male and female athletes. Specifically, although the most common reported cause of SRC in male athletes was contact with another player, accounting for almost half of SRCs recorded in males, in female athletes the most common mechanism was contact with an object (41.9%). These findings echo those reported in a meta-analysis^27^ of high school and college sports that revealed female soccer athletes were more likely to sustain an SRC from contact with a ball, including during heading, or equipment and less likely to sustain a concussion from player contact than male athletes. In general, soccer ball heading has been considered an infrequent direct cause of SRC,^28^ although it is widely recognized that collision with opponents during heading can result in concussion.^14^ Nevertheless, studies report evidence of acute cognitive^29,30^ and electrophysiologic disturbance^29^ and blood biomarker indexes of brain injury^31^ in the short term after a session of soccer ball heading and brain imaging changes during a season of exposure.^32^ Notably, research into the influence of sex on these outcomes is lacking; however, both male and female athletes may benefit from heading restrictions because of the frequency of player contact related to SRC in male athletes and equipment-related contact in female athletes reported in this study.

### Sex Differences in Concussion Management and Outcomes in Soccer

Standard of care in SRC management is immediate removal of the injured athlete from sport participation.^1^ However, this study found that delivery of this standard of care varied depending on who was delivering sideline medical assessment and between male and female athletes. When an athletic trainer was involved in the initial assessment, athletes with SRC were considerably more likely to be removed from play. Furthermore, male athletes were removed from play approximately 1½ times more often than female athletes. Research indicates that high schools with an athletic trainer have higher rates of recognized SRC,^33^ particularly in soccer.^34^ Continued play after an SRC is associated with increased symptom severity^35,36^ and length of recovery for athletes with SRC.^35,37,38,39,40^ Intriguingly, this study found that return-to-play time in female athletes was a median of 2 days longer than for male athletes. The possibility exists, therefore, that this longer recovery time might, in part, be reflective of our observed differences in immediate care, in particular removal from play.

### Limitations

This study has limitations. Although this was a large, statewide epidemiological study of reported SRC in adolescent soccer athletes, inclusive of high schools with and without access to athletic trainers, the Head Injury Reporting System did not include information on the whether there were athletic trainer services available at each school, including specific athletic training services for soccer. Evidence from the Athletic Training Locations and Services Annual Report^19^ suggests that in Michigan 45% of public high schools and 65% of private high schools do not have access to athletic training services. This report, coupled with the findings of the current study, should motivate future research to investigate the effect of athletic trainer employment on SRC risk and outcomes. A further limitation is that although the MHSAA regulates the total number of events an athlete can participate in, the Head Injury Reporting System does not include athlete exposures without injury events. Accordingly, only rates based on the total number of athletes participating each year are provided. There are also no details on wider clinical history for each soccer athlete; therefore, the association between premorbid or comorbid factors and SRC cannot be determined. Finally, to maintain deidentification in data collection procedures, the Head Injury Reporting System records each SRC as a new case. Thus, although prior concussion history is captured with each event history, there is no means to correlate this with and match to data on any previous reported SRC from the same athlete within the system.

## Conclusions

In this cohort study, marked sex-associated differences in reported SRC risk, management, and outcomes in soccer participants were revealed, suggesting that these populations could benefit from sex-specific game engagement. Congruent with previous literature,^6,8,9,10,11^ female athletes demonstrated a 1.8 times greater risk of documented SRC and took a median of 2 days longer to return to play compared with male athletes. Interestingly, the odds of immediate removal from activity were 3 times greater for reported SRC cases with an athletic trainer involved in the initial SRC evaluation, and male athletes had greater odds of immediate removal than female athletes. Furthermore, mechanism of injury differed between male and female athletes. Thus, these data suggest that risk reduction strategies for SRC in adolescent soccer might require sex-specific interventions, including measures to reduce game-associated risk such as sex-specific guidance on soccer ball heading and in the assessment of players involved in head injury events with potential for SRC.

## Notes

Supplement. **eFigure.** Kaplan-Meier Survival Curve Presenting the Proportion of Adolescent Soccer Athletes Not Authorized Clearance to Return to Unrestricted Activity
