# Supplementary material for: Association of Sex With Adolescent Soccer Concussion Incidence and Characteristics
Source: JAMA Netw Open. 2021 Apr 27;4(4):e218191. doi: 10.1001/jamanetworkopen.2021.8191 (PMC8080231; doi:10.1001/jamanetworkopen.2021.8191)
Supplement: Supplement. — eFigure. Kaplan-Meier Survival Curve Presenting the Proportion of Adolescent Soccer Athletes Not Authorized Clearance to Return to Unrestricted Activity [file jamanetwopen-e218191-s001.pdf]

Supplementary Online Content

Bretzin AC, Covassin T, Wiebe DJ, Stewart W. Association of sex with adolescent soccer concussion incidence and characteristics. *JAMA Netw Open*. 2021;4(4):e218191. doi:10.1001/jamanetworkopen.2021.8191

eFigure. Kaplan-Meier Survival Curve Presenting the Proportion of Adolescent Soccer Athletes Not Authorized Clearance to Return to Unrestricted Activity

This supplementary material has been provided by the authors to give readers additional information about their work.

© 2021 Bretzin AC et al. *JAMA Network Open.*

A.

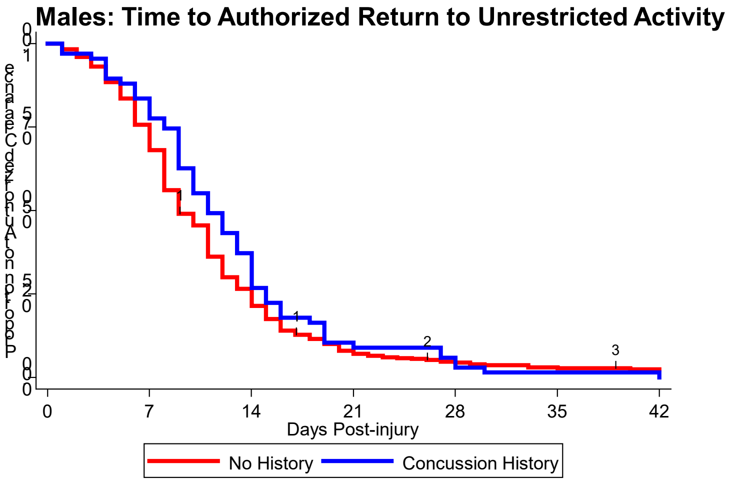

Number at Risk

Concussion History	67	56	25	7	4	2	1
No History	408	309	108	32	17	11	0

B.

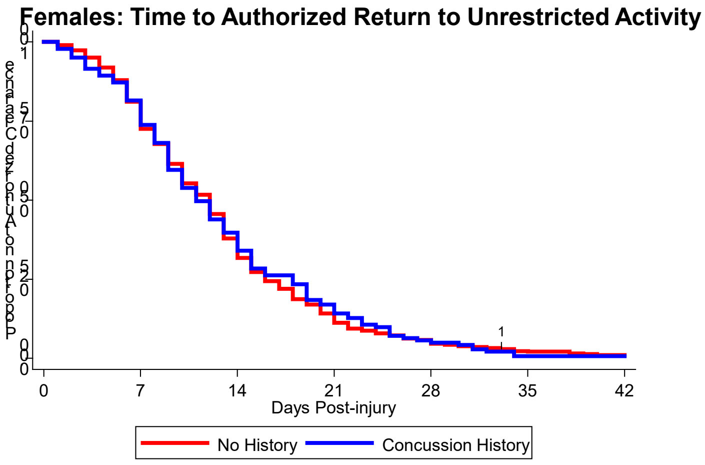

Number at Risk

Concussion History	141	115	56	24	8	3	1
No History	605	491	229	86	35	13	6

eFigure 1: Kaplan-Meier survival curve presenting the proportion of adolescent soccer athletes not authorized clearance to return to unrestricted activity.

A) Median time to return to activity for male athletes with a history of previous concussion was 11 (IQR 8 to 15) days compared to 9 (IQR 7 to 14) days for males without concussion history (p=0.023; Peto test **B)** Median time to return to activity for female athletes with a history of previous concussion was 11 (IQR 7 to 18) days compared to 12 (IQR 7 to 16) days for females without (p=0.928; Peto test).

© 2021 Bretzin AC et al. *JAMA Network Open.*
